# *Plasmodium knowlesi* gene expression differs in *ex vivo* compared to *in vitro* blood-stage cultures

**DOI:** 10.1186/s12936-015-0612-8

**Published:** 2015-03-13

**Authors:** Stacey A Lapp, Sachel Mok, Lei Zhu, Hao Wu, Peter R Preiser, Zybnek Bozdech, Mary R Galinski

**Affiliations:** Emory Vaccine Center, Yerkes National Primate Research Center, Emory University, Atlanta, GA USA; School of Biological Sciences, Nanyang Technological University, Singapore, Singapore; Department of Biostatistics and Bioinformatics, Rollins School of Public Health, Emory University, Atlanta, GA USA; Department of Medicine, Division of Infectious Diseases, Emory University, Atlanta, GA USA

**Keywords:** *Plasmodium knowlesi*, Malaria, Non-human primates, *Macaca mulatta*, Blood-stage infections, Microarrays, Antigenic variation, Gene expression, *SICAvar*, Haem

## Abstract

**Background:**

*Plasmodium knowlesi* is one of five *Plasmodium* species known to cause malaria in humans and can result in severe illness and death. While a zoonosis in humans, this simian malaria parasite species infects macaque monkeys and serves as an experimental model for *in vivo, ex vivo* and *in vitro* studies. It has underpinned malaria discoveries relating to host-pathogen interactions, the immune response and immune evasion strategies. This study investigated differences in *P. knowlesi* gene expression in samples from *ex vivo* and *in vitro* cultures.

**Methods:**

Gene expression profiles were generated using microarrays to compare the stage-specific transcripts detected for a clone of *P. knowlesi* propagated in the blood of a rhesus macaque host and then grown in an *ex-vivo* culture, and the same clone adapted to long-term *in vitro* culture. Parasite samples covering one blood-stage cycle were analysed at four-hour intervals. cDNA was generated and hybridized to an oligoarray representing the *P. knowlesi* genome. Two replicate experiments were developed from *in vitro* cultures. Expression values were filtered, normalized, and analysed using R and Perl language and applied to a sine wave model to determine changes in equilibrium and amplitude. Differentially expressed genes from *ex vivo* and *in vitro* time points were detected using limma R/Bioconductor and gene set enrichment analysis (GSEA).

**Results:**

Major differences were noted between the *ex vivo* and *in vitro* time courses in overall gene expression and the length of the cycle (25.5 hours *ex vivo;* 33.5 hours *in vitro*). GSEA of genes up-regulated *ex vivo* showed an enrichment of various genes including *SICAvar*, ribosomal- associated and histone acetylation pathway genes. In contrast, certain genes involved in metabolism and cell growth, such as porphobilinogen deaminase and tyrosine phosphatase, and one *SICAvar* gene, were significantly up-regulated *in vitro*.

**Conclusions:**

This study demonstrates how gene expression in *P. knowlesi* blood-stage parasites can differ dramatically depending on whether the parasites are grown *in vivo*, with only one cycle of development *ex vivo,* or as an adapted isolate in long-term *in vitro* culture. These data bring emphasis to the importance of studying the parasite, its biology and disease manifestations in the context of the host.

**Electronic supplementary material:**

The online version of this article (doi:10.1186/s12936-015-0612-8) contains supplementary material, which is available to authorized users.

## Background

*Plasmodium* invasion, growth and development in red blood cells (RBCs) is a complex process entailing many levels of controlled gene expression, receptor-ligand interactions, host cell modifications, and communication with the outside milieu. Each species of *Plasmodium* has between 5,100 and 7,700 or so genes [1], which as first shown for *Plasmodium falciparum,* become differentially expressed throughout the parasite’s intra-developmental cycle (IDC) in the blood [2-4]. Understanding the multitude of molecular and cell biological events required by *Plasmodium* to successfully parasitize RBCs and evade host defenses is paramount and increasingly within reach with post-genomic approaches [5-7].

The development of a continuous *in vitro* blood-stage culture system for *P. falciparum* was an essential step towards significantly advancing research in many of these areas of blood-stage malariology, as well as the development of malaria diagnostics, drug and vaccine candidate interventions [8]. A comparably robust blood-stage culture system is also available for the simian malaria species *Plasmodium knowlesi* [9,10], but not other primate malaria species. Attempts have been made to develop continuous cultures of *Plasmodium vivax* [11] and the very closely related simian malaria parasite species *Plasmodium cynomolgi* [12], but the preference for reticulocyte host cells along with other possible requirements that have yet to be discovered have so far prohibited the development of routine long-term culture systems for these species [13-15].

While the utility of *P. falciparum* and *P. knowlesi in vitro* blood-stage culture systems has been evident for many avenues of research, including genetic manipulation to study gene expression characteristics and determine functional traits, the biological states of parasites in host organisms can differ substantially from that of *in vitro* cultured parasites. For example, this became evident for *P. falciparum* with recognition of the loss of non-essential gene segments in culture [16] and reviewed in [17]. Expression of the variant antigen *var* gene transcripts from *P. falciparum* have also been shown to become down-regulated when clinical isolates are adapted to *in vitro* culture [18,19]. Numerous changing metabolic and physical parameters in the parasite and host factors are either different or lacking *in vitro,* which may affect the parasite’s normal growth and biology. Investigations have shown that even one altered parameter, such as glucose level, can result in a marked change in gene expression profiles in cultured *P. falciparum,* with 560 genes exhibiting a two-fold or greater change in the presence of 0.6 mM glucose compared to 11.1 mM glucose [20].

Notably, *P. knowlesi* can readily be studied *in vivo, ex vivo* and *in vitro,* and it can potentially serve as a model for *P. falciparum* or *P. vivax;* depending on the scientific question. *Plasmodium knowlesi* is currently also recognized as a species that can be transmitted to and cause illness and death in humans [21-23]. Given its broad utility, many critical basic malaria research discoveries have been made using *P. knowlesi*. These include classic investigations of merozoite attachment to and invasion of erythrocytes after the formation of a tight junction [24,25], the first report of the phenomenon of antigenic variation (in rhesus monkeys) [26], and the first identification of parasite-encoded variant antigens expressed at the surface of infected RBCs; known as the Schizont Infected Cell Agglutination (SICA) antigens [27]. The SICA antigens are encoded by a large multigene family known as *SICAvar* [28-30], which is functionally analogous and evolutionarily related to the *var* gene family in *P. falciparum* [31,32]; both gene families are highly expressed in ring stages [28,30,33]. Expression of the SICA proteins *in vivo* in rhesus monkeys is associated with the increased virulence of *P. knowlesi*, and both the expression of these proteins and switching of variant types at the surface of the infected host cells is dependent on the presence of the spleen [34]. Recent studies demonstrate for the first time that SICA[−] parasites lack full-length *SICAvar* transcripts and lack SICA protein, consistent with apparent post-transcriptional processing and translational repression [30].

Particularly now with the expanding potential for post-genomic exploration of *Plasmodium* biology and pathogenesis, and the use of comprehensive systems biology approaches to understand host-pathogen interactions, it is important to define and be cognizant of the similarities and differences in gene expression that may occur when *Plasmodium* is grown in a living host, compared to *in vitro*. Here, these parameters are examined by studying the stage-specific gene expression profiles of *P. knowlesi* blood-stage parasites when grown in a rhesus monkey followed by one cycle of *ex vivo* growth and development compared to long-term *in vitro* culture. The majority of the observed microarray signals correspond to genes that are annotated in the current *P. knowlesi* genome database as hypothetical. Of the various differentially expressed (DE) known genes, it is striking that signals representing a large number of *SICAvar* gene transcripts were strongly up-regulated in the *ex vivo* (ring-stage) samples, but not in the *in vitro* samples. These observations *in toto* are germane for understanding the biology of the *P. knowlesi* intraerythrocytic development cycle (IDC) and the pathogenesis and virulence of this species. The results are also highly pertinent as indicators of what biological or biochemical networks and pathways may likewise be affected when other species including *P. falciparum* are grown and propagated long-term under *in vitro* conditions.

## Methods

### Ethics statement

Animal experimentation reported here involved one animal (*Macaca mulatta*; rhesus macaque) and was conducted in AAALAC-accredited facilities at the Yerkes National Primate Research Center of Emory University in accordance with the Animal Welfare Act and the Guide for the Care and Use of Laboratory Animals. Emory University’s Institutional Animal Care and Use Committee (IACUC) approved this project’s experimental protocol. Temperatures in animal areas are controlled and monitored by an automated on-line system. The system has set parameters for high and low temperatures, maintained at 22°C ± 3°. The photoperiod is set at a 12/12 cycle. Rhesus macaques are housed singly, and provided with one of the following: forage board, peanut feeder, challenger ball, or other similar device for enrichment. In addition, destructible enrichment is distributed as prescribed to monkeys. Rhesus macaques receive jumbo biscuits (15% protein). Animals are fed twice daily. The amount of food is adjusted depending on species, sex, age, weight and specific number of animals living in a group. Consumption is monitored and adjustments are made as necessary so that animals get enough food with minimum waste. Enrichment produce is fed twice weekly. All animals are monitored daily by veterinary staff for potential health problems. Experienced personnel trained all animals to voluntary present at the front of the cage for skin prick using positive reinforcement. For blood collection, as required by this study, animals are sedated with Ketamine (10 mg/kg IM), which is allowed no more than twice per 24 hours. Per IACUC guidelines, maximum blood volume collected cannot exceed 10 ml/kg/month. The one rhesus macaque assigned to this study was treated and available for other studies at the Yerkes National Primate Research Center.

### Parasite samples for microarray analysis

An *ex vivo P. knowlesi* blood-stage culture was initiated using iRBCs from a rhesus macaque infected with the Pk1(A+) clone of *P. knowlesi* [27]. The Pk1(A+) clone was selected since the genomic DNA from this clone was used to develop the first reported *P. knowlesi* genome sequence [35]. Briefly, a cryopreserved vial of ring-stage parasites was thawed, processed and inoculated into a rhesus monkey using standardized procedures. Parasitaemia was monitored daily and whole blood was collected into heparin-treated tubes when it reached 8% ring-stage infected RBCs (iRBCs). The monkey was treated with 15 mg/kg of chloroquine for 3 days to clear the parasites. Collected whole blood was treated with a final concentration of 2 mg/ml ADP (adenosine diphosphate bis salt) for 5 min before passage through a glass bead column equilibrated with Hank’s balanced salt solution to remove platelets, followed by passage through a Plasmodipur filter. The filtered blood was centrifuged for 5 min at 800 g and the pellet resuspended in RPMI with L-glutamine, supplemented with 0.25% sodium bicarbonate, 50 ug/ml hypoxanthine, 7.2 mg/ml HEPES, 2 mg/ml glucose, 10% human AB+ serum. The iRBCs were cultured at 1 × 10^7^ iRBCs/ml in multiple 175 cm^2^ flasks (one per time point, TP) and gassed with 5%: 5%: 90%, O2:CO2:N2. One culture flask was processed every four hours over a 24-hour period for extraction of parasite RNA, and these samples were designated as *ex vivo* TP #s 1–6.

For the *in vitro* time course samples, a cryopreserved vial of culture-adapted Pk1(A+) parasites was thawed using standardized procedures, and expanded in RPMI with L-glutamine, supplemented with 0.25% sodium bicarbonate, 50 ug/ml hypoxanthine, 7.2 mg/ml HEPES, 2 mg/ml glucose, 10% human AB+ serum. Fresh rhesus RBCs were added as necessary, and the parasitaemia was maintained at less than 10%. The culture was synchronized by consecutive Percoll (52%) and sorbitol treatment in which RBCs were suspended in 5X volume of pre-warmed 5% sorbitol, incubated for five minutes and washed three times in RPMI [36]. Since the *in vitro* cultured parasites develop at a slightly slower rate than *in vivo*, a 28- or 32-hour time course plan was designed for RNA extraction, with iRBC collection every four hours. Samples were generated representing two biological replicates (I and II), with samples for seven TPs collected for the first replicate and eight for the second; the eighth TP was particularly aimed at collecting very mature segmenting schizonts.

### RNA extraction

For the first two time points from the *ex vivo* culture, 1 × 10^10^ iRBCs were collected and put into Trizol LS (Invitrogen) at a 1:10 pellet to reagent ratio, vigorously shaken for 1 min, incubated at 37°C for 5 min, and stored at −80°C. For all other time points from the *ex vivo* culture, 5 × 10^9^ iRBCs were collected. At the last time point, a 1:20 pellet to Trizol LS ratio was used to extract RNA. All samples were immediately stored at −80°C. For the *in vitro* cultures, approximately 5 × 10^9^ parasites were collected for the first two time points, and 1 × 10^9^ parasites were collected for the remaining time points. To purify the total RNA, the parasite/Trizol samples were thawed at room temperature and transferred to 30 ml Teflon tubes. One ml chloroform per 5 ml sample volume was added and vigorously shaken for 1 min before centrifuging at 12,000 g for 30 min at 4°C. The aqueous layers were removed to a 30 ml polypropylene tube, 0.8 volume of isopropanol added, and incubated overnight or for at least 2 hours at −20°C before centrifuging at 12,000 g for 30 min at 4°C. The supernatant was carefully removed and the pellet was allowed to dry for 5–10 min at room temperature and then resuspended in 1 ml of RNase-free water. The sample was purified using the RNeasy midi kit (Qiagen) following the manufacturer’s instructions.

### Proteomic analysis

Briefly, late stage trophozoites from synchronized cultures were purified with 52% percoll, washed 3 times with incomplete RPMI 1640, resuspended in SDS sample buffer and stored at −80°C. Immunoprecipitation with a pan-SICA antibody was performed as in [30]. Peptides were generated as previously described [31]. Protein bands were excised from SDS-PAGE gels and gel pieces destained and dried. Dried gel bands were digested at 37°C overnight with 0.4 g of proteomics grade trypsin (Sigma, St. Louis, MO, USA). The resulting peptides were then extracted with 0.1% trifluoroacetic acid in 50% acetonitrile (Sigma), desalted and concentrated using ZipTip pipette tips containing C18 reversed-phase media (Millipore, Billerica, MA), and then washed in 0.1% trifluoroacetic acid and eluted in 0.1% trifluoroacetic acid/50% acetonitrile (Sigma). Cleaned peptides were analysed by reverse-phase LC-MS/MS using an LTQ-Orbitrap mass spectrometer (Thermo Finnigan). A reverse database strategy using the SEQUEST algorithm was implemented to evaluate false discovery rate; the matched peptides were filtered according to matching scores to remove all false matches from the reverse database [37]. Only proteins that were matched by at least two peptides were accepted to further improve the confidence of identification. The peptides were then searched against the NCBI database, with searches being limited to *Plasmodium* results. The *P. knowlesi* identified protein sequences were then used to BLAST (Basic Local Alignment Search Tool) against the NCBI database for sequence homologies.

### Data analysis

#### Reannotation of oligonucleotide probes

A set of 60-mer oligonucleotide probes (Additional file 1) developed prior to the publication of the *P. knowlesi* genome was reannotated based on the genome sequence reported in PlasmoDB V8.2. All the oligonucleotides in the array were BLASTed against the PlasmoDB V8.2 sequences using NCBI BLAST 2.2 with the parameter of E-value cutoff at 0.1. Only oligonucleotides with unique hits to protein-coding regions were kept for further analysis. The unique hit oligonucleotides were defined as probes having high first-hit bit scores (>85) and low second-hit bit scores (<60) or probes having second-hit bit scores equal to 0. Of the 4,799 *P. knowlesi* probes found to have unique hits, 4,755 representing 4,700 protein-coding genes were used in the analysis.

### Determining the periodicity of gene expression

#### Microarray hybridization, data filtering and normalization

All data were processed using R and Perl language. The workflow is shown in Additional file 2. cDNA synthesis, Cy dye labelling, as well as microarray hybridizations of the time course samples were carried out as described previously [38]. For each *P. knowlesi* time course, sampling time point RNAs labelled with Cy5 were hybridized against a reference RNA pool labelled with Cy3 (consisting of equal amounts of *P. knowlesi* RNA from each time point). Microarray hybridizations were carried out on a chip with 4,992 60-mer oligonucleotides representing 4,700 coding genes designed for *P. knowlesi*, with the remainder representing *P. vivax* genes*.* Microarray data acquired with GenePix Pro 6.0 software (Axon Instruments, USA) were filtered for quality control by accepting only features with Cy3 and Cy5 signal intensities higher than two-fold of the microarray background intensities. The log2 ratio of Cy5/Cy3 intensity was calculated after background subtraction and was normalized using locally estimated scatterplot smoothing (LOESS) in *marray* package of R. To reduce technical noise between experiments, robust quantile normalization was applied to the data across all time points to achieve the same distribution of log2 ratios (*Affy* package of R). After normalization, probes were filtered out that had more than two missing values across all time points for any one of the three time courses, resulting in 4,198 probes, representing 4,165 protein-coding genes that were used for further analysis (Additional file 3). The microarray probe ratios were averaged for multiple probes of a particular gene. Data for the microarrays and probe information are available at the Gene Expression Omnibus database at the National Center for Biotechnology Information [GEO: GSE62886, GPL19374].

### Sine wave modelling

**Figure 1:**
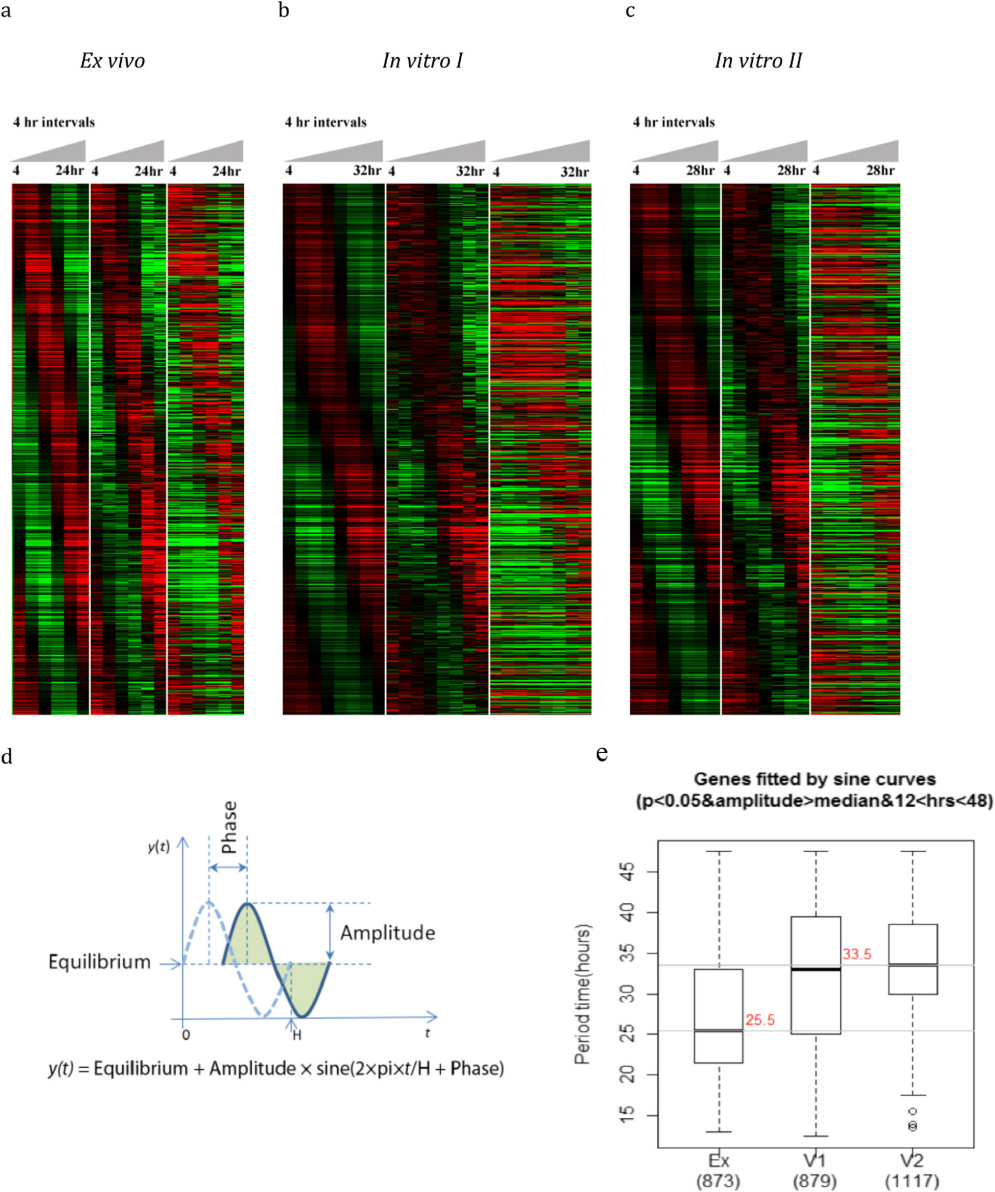
**Periodical transcription of** ***P. knowlesi*** **genes during the IDC.** Transcriptomes showing 4,165 PK genes of the *ex-vivo* **(a)** and two *in-vitro* samples **(b,c)** of the time course sampled at 4 hour intervals over 24–32 hours in culture. First column represents raw data during time courses; second column represents raw data after median centered normalization and third column represents equilibrium value centered data after smoothing by sine functions with H = 27 for *ex vivo* and H = 33 for *in vitro*. **(d)** Sine wave model and its equation. *t* is the time (hours) at each time points, and *y(t)* is the corresponding transcription levels at each time point. Equilibrium is the average level of one complete sine curve. Amplitude is visually represented as the vertical distance between the extrema of the sine curve and the equilibrium value. *h* is the period of one complete life cycle expressed in hours and phase is the fraction of one life cycle indicating time offset of an expression peak to the time point at one quarter of life cycle. **(e)** Prediction of duration for a complete cycle of *ex vivo* and *in vivo* samples (period time). 873 genes from *ex vivo* sample, 879 and 1117 genes from *in vivo* samples I and II significantly fit a sine curve (p value < 0.05 and amplitude above median) and were used to predict the period. The median period is 25.5 hours for *ex vivo* sample, and 33.5 hours for *in vitro* samples.

To compare gene transcription between the iRBC samples without knowing the exact time post-invasion, a sine wave model was applied. The function is described in Figure 1d. *t* is the time (hours) at each time point, and *y(t)* is the corresponding transcription levels at each time point. Equilibrium is the average level of one complete sine curve. It was assumed to be the balanced transcription level of a gene across the life cycle, an original state that a gene would return after expression peak, valley and any expression changes. Amplitude is the transcription level change according to equilibrium, visually represented as the vertical distance between the extrema of the sine curve and the equilibrium value. *h* is the period of one complete life cycle expressed in hours and phase is the fraction of one life cycle indicating the time offset of an expression peak to the time point at one quarter of the life cycle. For each gene, the parameters equilibrium, amplitude, and phase were determined and *h* optimized within 12 to 48 by the sine wave model to find a sine function that best fits the periodical pattern of gene transcription levels across the life cycle.

### Phase adjustment

Phase is the initial angle of a sinusoidal function at its origin. It indicates the time offset of an expression peak to time zero. The magnitude of the phase correlates to the timing of the peak expression of a gene. Since it is difficult to synchronize cells at the exact same starting time, the calculated phase for the same gene from time course to time course would differ. Therefore, phase adjustment is required to adjust for these differences. Using the established sine wave model, expression levels were estimated for each gene at each hour during its life cycle to give rise to 25 time points (*ex vivo*) and 33 time points (*in vitro*). The *in vitro* time points were mapped to *ex vivo* time points by determining the highest correlated time point between *ex vivo* and *in vitro* expression profiles (Additional file 4). To make the result more reliable, only 219 genes having significant periodical expression patterns were used (p value < 0.05 and amplitude >0.3 of their sine curve) in all three samples. Phase shift was estimated for each *in vitro* time point based on the corresponding *ex vivo* sample. The average of phase shift then was used as the delta phase, 0.85 between *in vitro* I and *ex vivo*, 1.4 between the biological replicate *in vitro* II and *ex vivo*.

### Functional enrichment analysis

Gene set enrichment analysis (GSEA) was carried out based on GO terms, KEGG pathway and MPM pathway annotated for each *P. knowlesi* gene according to their *P. falciparum* orthologs (PlasmoDB v8.2) [39]. 4,570 of 5,197 protein-coding genes in *P. knowlesi* have orthologs in *P. falciparum* (3,838 *P. knowlesi* genes with at least one GO term, 761 genes with at least one KEGG identification, and 2,346 genes with at least one MPM identification). In the gene enrichment analysis, only functional groups with more than 10 genes were considered. In addition, another eight functional groups were added including *SICAvar*, *kir* and others derived from recent work [40].

### Differential expression analysis by time point

For each microarray, logarithms of fold changes between the various experimental time point sample RNAs and reference RNAs were first computed for all genes. Then cross-array normalization was applied to remove global technical biases brought by different arrays. For each array the log-fold changes were shifted and rescaled so that they have the same means and variances. There are expression values for each gene at six time points for the *ex vivo* time course and seven time points for the two *in vitro* time courses. To make the data comparable for different samples, the seven expression values for the *in vitro* samples were mapped into six time points by interpolation (Additional file 5).

### Assessing global differences in gene expression among the three time courses

To assess the global differences in gene expression among the *ex vivo* and *in vitro* samples, the pair-wise differences of expression were computed among the three samples for all genes. For a gene, the difference score between two samples is defined as the Euclidean distances for its expression at all time points. Smaller distance means greater similarity. By doing so three difference scores were obtained for each gene, whereby each represents the difference between a pair of samples. For a gene, if the difference score between the two *in vitro* samples is the smallest among the three, it indicates that the expression of that gene is more similar between the *in vitro* samples.

To rigorously assess the significance of this proportion, a randomization test was performed. In each permutation, gene labels were randomly shuffled for all samples but the time points were kept unchanged. Then the pairwise difference scores were recomputed for all genes, and the number of genes with difference scores between the *in vitro* samples being the smallest was computed.

### Detecting individual differentially expressed (DE) genes

To detect individually DE genes from *ex vivo* and *in vitro* samples, the two sets of *in vitro* samples were treated as biological replicates. DE genes at each time point were detected using limma R/Bioconductor software package [41]. To detect DE genes considering data from all time points, a functional *t*-test procedure was adopted [42]. Specifically, for each gene a modified t-statistic was computed at each time point, and then the maximum of the t-statistics from all time points was obtained as the test statistics for the gene. The critical value for differential expression can be obtained through a permutation test. Because of the small sample size, stringent criteria were used and the maximum of all t-values from all permutations were used as the threshold for defining DE genes.

## Results and discussion

Microarray experiments were performed to evaluate the global gene expression profiles of *P. knowlesi* blood-stage parasites as they mature in host red blood cells, from ring to trophozoite and schizont stages of development, and compare the profiles between *ex vivo* cultures and *in vitro* adapted cultures. An overarching purpose of this research was to determine which gene expression patterns if any may be dependent on the presence of host factors.

A *P. knowlesi* infection was established in a naïve rhesus monkey and the maximum blood volume allowed by IACUC guidelines and approved protocols (10 ml/kg/month) was drawn to obtain ring-stage iRBCs for *ex vivo* culture and the collection of stage-specific samples every 4 hours over the parasite’s 24-hour IDC. *Plasmodium knowlesi* asexual stage development is synchronous *in vivo,* making this readily feasible, beginning with ring-stage iRBCs collected from a blood draw. It would have been ideal from a scientific standpoint to obtain blood collections throughout the IDC, but, per IACUC guidelines, animals may only be sedated twice/day, and in addition to logistical limitations of the availability of animal resource staff over a 24-hr period, *ex vivo* cultures were by necessity initiated when the iRBCs were at the ring stage of development.

For comparison, two biological replicates were generated from *in vitro* adapted cultures, which do not maintain synchronous growth [9]*.* Acquiring these samples for comparison with the *ex vivo* cultures, therefore, required synchronization steps (see Methods). The first *in vitro* time course was collected over 28 hours. For the second *in vitro* time course an additional sample TP was collected at 32 hours to obtain segmented schizonts, the final stage of the parasite’s growth before the release of merozoites. These cultures were maintained by the addition of freshly acquired RBCs from a different rhesus macaque than the one used for the *ex vivo* time course; in our view the specific host RBCs themselves would have negligible impact on the transcription profiles over one cycle compared to the culture environment (*ex vivo* versus long term *in vitro*). Notably, the Pk1(A+) line is routinely grown in culture medium supplemented with human AB+ serum. An overarching goal of this work is to compare parasite biology when adapted to widely available long-term culture conditions versus *ex vivo*. For this reason and consistency through multiple experiments, human AB+ serum pooled from 10 different donors was used in lieu of rhesus macaque serum.

### Periodicity of gene expression in the *P. knowlesi* IDCs

To test for periodicity of gene expression in the *P. knowlesi* IDCs, sine wave regression was carried out for each expressed gene during the established time courses. 3,684 (~88.5%) out of the 4,165 genes examined on the array significantly fit to a sine curve with a p value <0.05 (F test) for at least one of the time courses. These analyses confirm that a majority of the parasite’s genes are transcribed periodically during the blood-stage cycle (Figure 1a, b and c), and each gene’s transcriptional profile can be fit to a sine wave with a determined equilibrium, amplitude and frequency (Figure 1d). For each time-course experiment, the time required for the parasite to complete the blood-stage cycle of growth and development was estimated. For each gene, varying the cycle length from 12 hours to 48 hours identified the best-fit sine curve, and the optimized sine function with the minimum F test p-value was gained. Based on this assumption, the length of each blood-stage cycle was estimated from genes that were significantly fitted by a sine curve (p value < 0.05) and amplitude above the median (based on 873 genes for the *ex vivo* time course, and 879 and 1,117 genes for the *in vitro* I and II time courses, respectively).

Parasites grown under *in vitro* conditions developed at a significantly slower rate (25 hours *ex vivo* versus 33 hours *in vitro*) (p value < 2.2e-16) (Figure 1e). Sine wave models were therefore established separately with the periodic time as 25 hours and 33 hours for the *ex vivo* and *in vitro* samples, respectively. Smoothed transcription profiles are shown in the second columns of the heat maps in Figure 1a, b and c. In addition, the age of the parasite at the start of the TP collections (TP1) was markedly different for each time course when the pair-wise correlation of TP1 was calculated between the time courses. This is a reflection of the inherent difficulty in precisely matching the ages of ring-iRBCs acquired directly from a rhesus macaque infection with those from synchronized *in vitro* cultures. Hence, data processing and adjustments were necessary to compare the *ex vivo* and *in vitro* transcriptomes.

### Comparative overview of the transcriptomes

**Figure 2:**
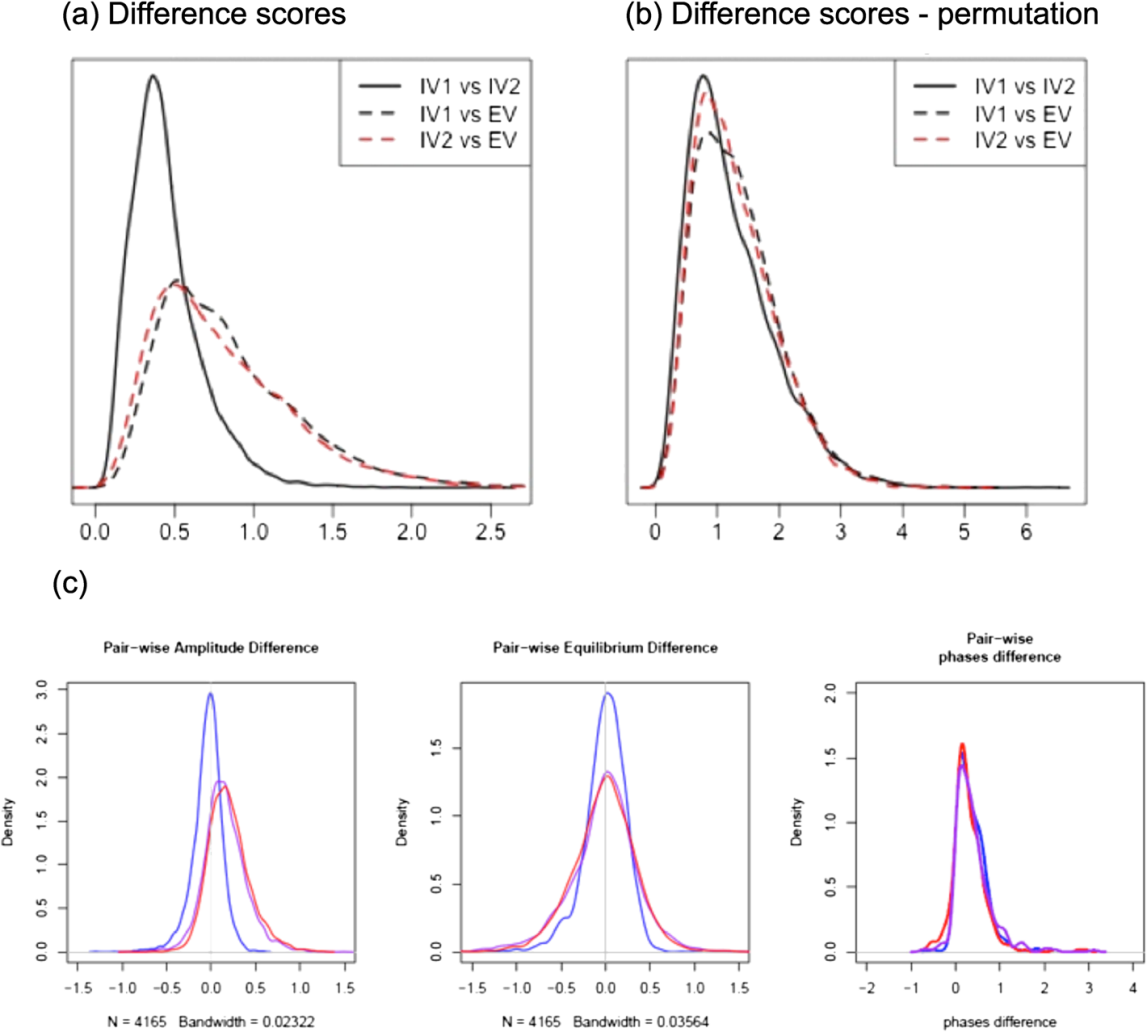
**Overall differences between** ***in vitro*** **and** ***ex vivo*** **time courses. (a)** The distributions of pair-wise difference scores for all genes showing the similarity of the two *in vitro* experiments and how they differ from the *ex vivo* experiment **(b)** the distributions of pair-wise difference scores from a permutation showing that the difference scores do not occur by chance. **(c)** Comparison of amplitude, equilibrium and phase between *ex vivo* and *in vitro* samples. These plots show the distribution of pair-wise difference of amplitude, equilibrium and phase values of *ex vivo* and *in vitro* I (red), *ex vivo* and *in vitro* II (pink), and *in vitro* I and *in vitro* II (blue). The amplitude difference was defined between *ex vivo* and *in vitro* as the minimum difference between *ex vivo* and the two *in vitro* named as D-EV_amp_ and the amplitude difference between the two *in vitro* samples as D-VV_amp_. The equilibrium difference (D-EV_eqm_ and D-VV_eqm_,) and phase difference (D-EV_ph_ and D-VV_ph_) were similarly defined.

Using the sine wave model, DE genes between the transcriptomes were defined based on equilibrium, amplitude and phase parameters as calculated for each gene. Distinctive gene expression differences were not noted between the two *in vitro* transcriptomes by these methods, or others, and pair-wise differences of gene expression among the three time courses (*ex vivo*, and the two *in vitro* biological replicates) indicate that 77% of the expressed genes are in fact more similar between the *in vitro* time courses (Figure 2a). In a randomization test based on 1,000 permutations, the permutation p-value was determined to be less than 0.001. These results strongly indicate that the gene expression profiles derived from the two *in vitro* time courses are much more similar to each other compared to the gene expression data from the *ex vivo* samples (Figure 2b). The pairwise differences in amplitude (p value < 2.2e-16) and equilibrium (p value < 2.2e-16) are significantly smaller between *in vitro* samples than between *in vitro* and *ex vivo* samples. In addition, the amplitudes of gene expression were found to be significantly higher for the *ex vivo* TPs than the *in vitro* TPs (p value < 2.2e-16), which is likely a result of the greater degree of synchronicity of the *ex vivo* culture. In the phase analyses, as expected, no significant changes were observed among the *ex vivo* and *in vitro* samples (Figure 2c).

### Differentially expressed genes in equilibrium and amplitude

**Figure 3:**
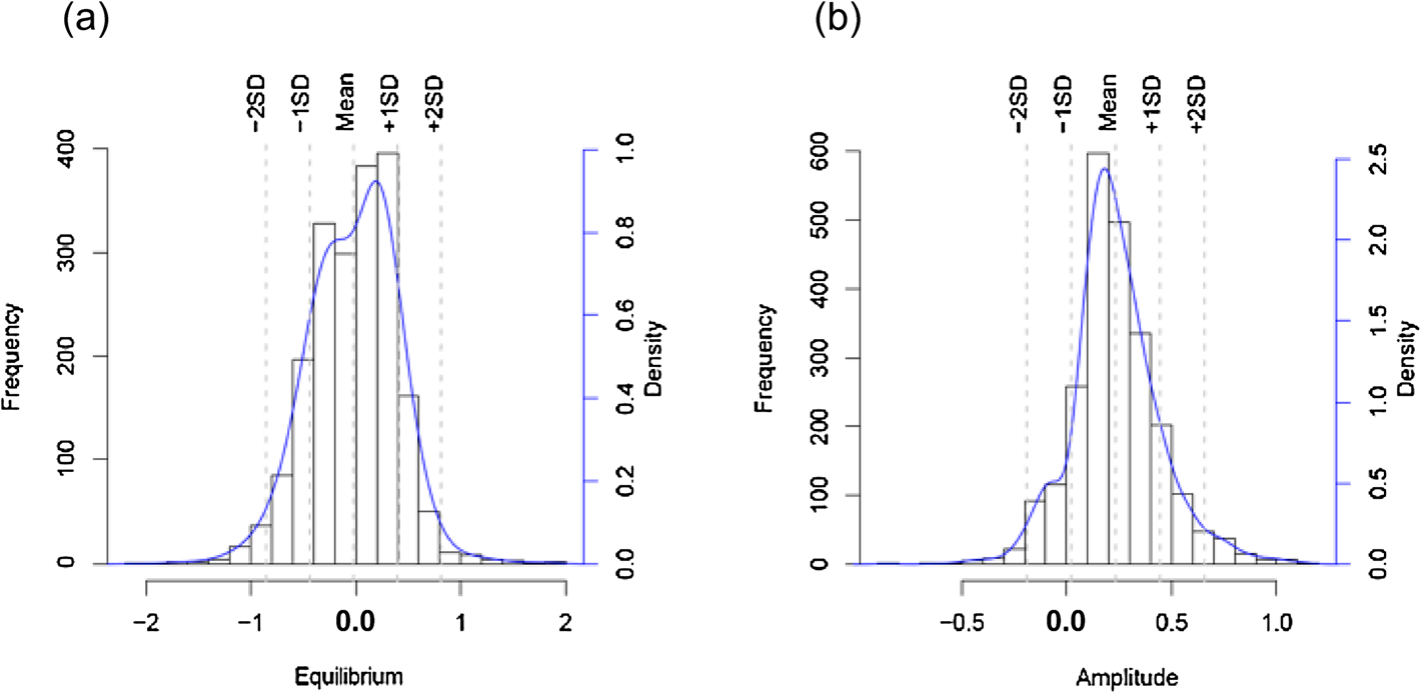
**Histogram showing the frequency of DE genes in terms of fold change between** ***ex vivo*** **and** ***in vitro*** **samples with respect to amplitude and equilibrium (log**_**2**_ **scale). (a)** Number of genes differentially expressed in equilibrium at each fold change cutoff **(b)** Number of genes differentially expressed in amplitude at each fold change cutoff. The majority (~89%) of genes were up regulated.

Differentially expressed genes were defined in equilibrium and amplitude based on the criterion that the minimum differences among the *ex vivo* and each of the two *in vitro* cycles were greater than the differences noted from the *in vitro* replicates. Taking all 4,165 considered genes into account, 2,009 transcripts were identified with changes in equilibrium and 2,355 transcripts with changes in amplitude. The distributions of gene numbers at each fold change are shown in Figure 3. GSEA was performed as described using *P. knowlesi* orthologs of *P. falciparum* genes belonging to annotated functional gene sets/pathways from KEGG, GO and MPM databases [43-45], in addition to several *P. knowlesi* specific gene sets including the predominant large *SICAvar* and *kir* multigene families*.*

**Figure 4:**
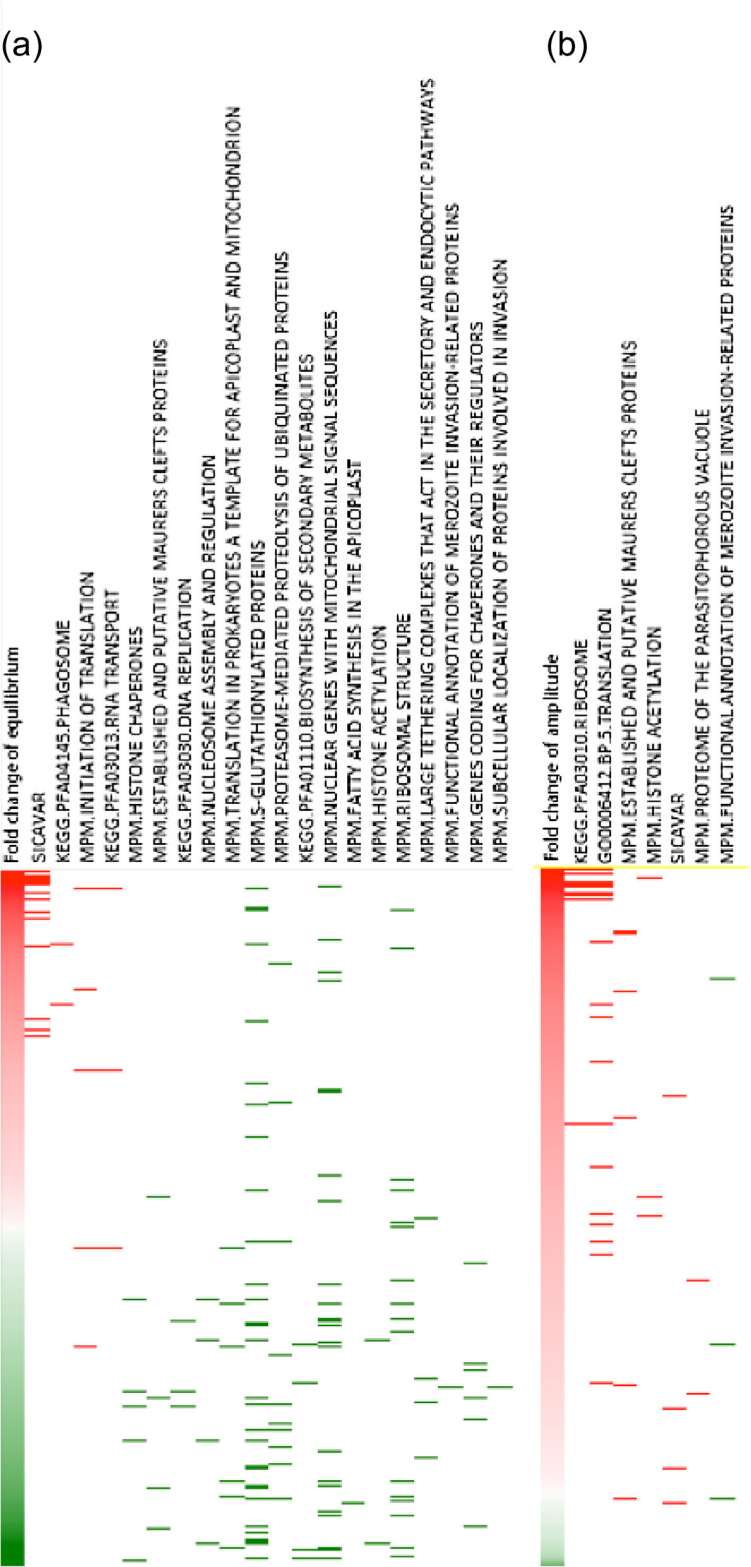
**Functional enrichment analysis of DE genes using GSEA software. (a)** Significantly enriched functional groups for genes differentially expressed at equilibrium levels (FDR < 25%, p value <0.05) **(b)** Enriched functional group of DE genes in amplitude (FDR < 25%). Red color represents up regulated genes. Green color represents down regulated genes. The short lines indicate genes from the corresponding functional pathway that is shown in the header.

Importantly, among the 1,029 (~51%) genes that were up-regulated in equilibrium in the *ex vivo* samples, there was significant enrichment in genes coding for SICA proteins (FDR = 0). Other genes noted in this category are associated with biological processes relating to RNA transport, translation initiation, and phagosomes. Conversely, 980 (~49%) of the genes that were down-regulated in equilibrium showed enrichment in transcripts involved in multiple biochemical pathways associated with nucleosome and chromatin assembly and organization, as well as DNA replication, proteasome-mediated proteolysis of proteins, and Maurer’s cleft related proteins (FDR < 25%, Figure 4a). Genes associated with ribosomal structure and fatty acid synthesis and certain organellar specific proteins (plastid, mitochondrion and ER) are among those that were less so, but also noticeably down-regulated in the *ex vivo* culture.

A majority (2,108 or ~89%) of genes with changes in amplitude were up-regulated in the *ex vivo* cultured parasites. These genes mainly include ribosomal associated genes, *SICAvar* genes, Maurer’s Cleft related protein genes and histone acetylation pathway genes, while the down-regulated genes (247, ~11%) showed an enrichment to just a few invasion-related genes (FDR < 25%, Figure 4b). As a whole, the results reveal that the *ex vivo* parasite has an overall higher capacity for transcribing certain genes involved in the interaction of host-parasites with larger stage-specific amplitudes, an effect apparently resulting directly from the *in vivo* growth of the parasite in rhesus monkeys, and in support of predictions to this effect. The up-regulated genes are accompanied by lowered transcription of various metabolic pathway genes such as for DNA replication, protein degradation and chromatin organization.

Significant DE genes were defined by applying the criterion that the minimum difference between the *ex vivo* and each of the *in vitro* cycles was greater than 1.5-fold (equal to Mean ± ~1.5 SD) of the difference between the *in vitro* replicates. 95 and 130 genes were up-regulated, and 162 and two genes were down-regulated, in equilibrium and amplitude, respectively.

### Differentially expressed genes by stage specificity

**Figure 5:**
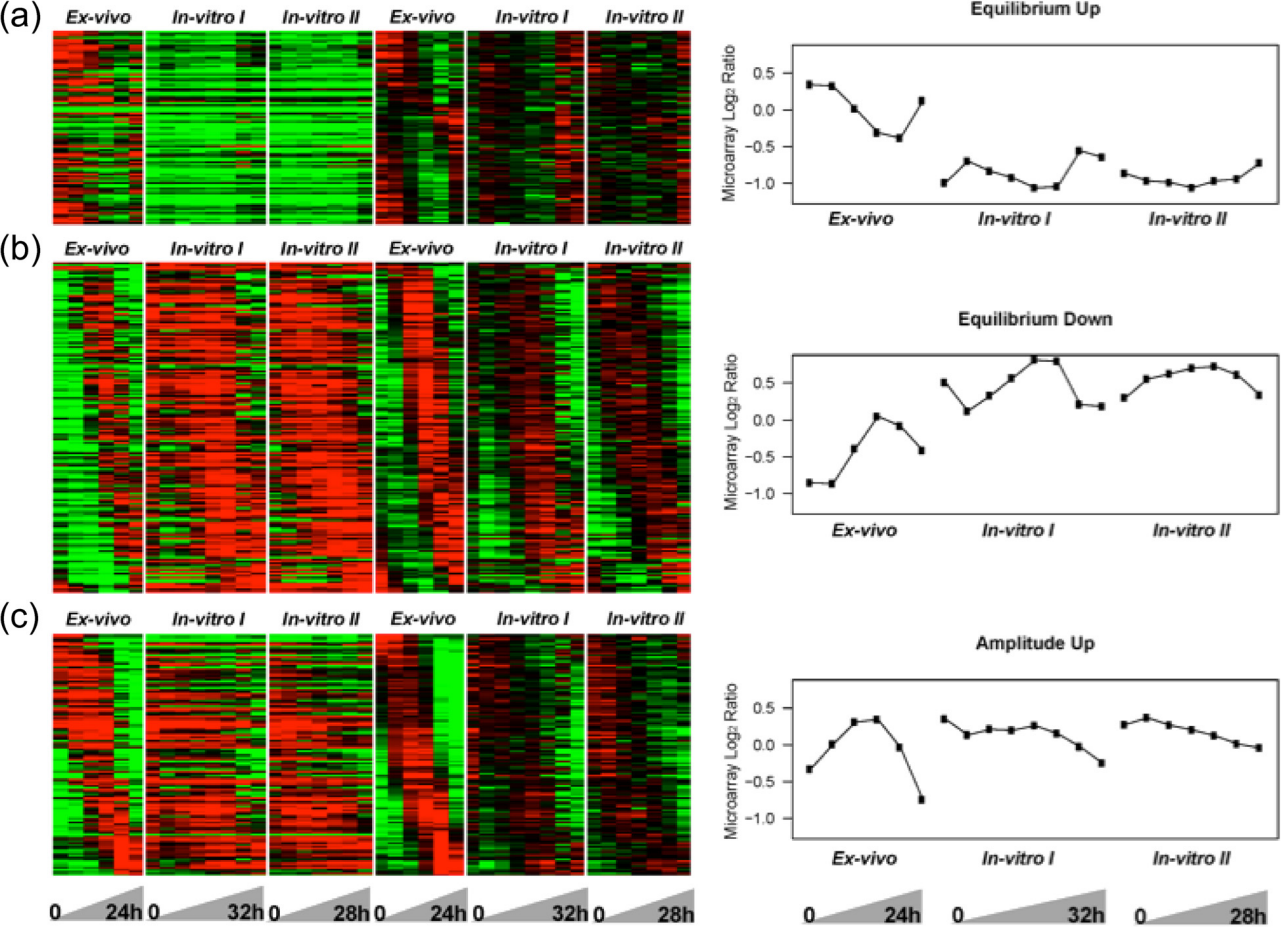
**Average transcription profile and heatmap of DE genes during time courses (fold change > 1.5). (a)** 95 genes up-regulated in equilibrium **(b)** 162 down regulated in equilibrium **(c)** 130 up-regulated in amplitude.

To determine if the DE genes were biased towards any particular stage in the IDC, the transcription profiles of all DE genes were averaged, as shown in Figure 5a, b and c. Genes that were up-regulated in equilibrium in the *ex vivo* culture were most highly expressed in the early ring stage and late stage schizonts (Figure 5a), while genes that were down-regulated in the *ex vivo* culture had peak expression in the trophozoite and early (2–4 nuclei) schizont stages (Figure 5b). Similarly, genes that were up-regulated in amplitude in the *ex vivo* culture were also highly expressed in the trophozoite stage (Figure 5c). The major significantly enriched functional pathways for the DE genes had peak expression that coincided with the stage of peak expression observed here.

**Figure 6:**
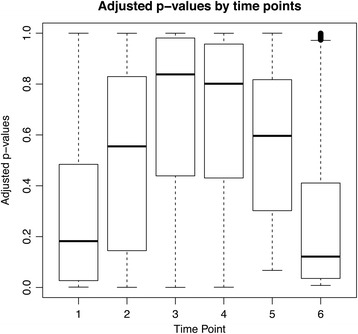
**Box plot showing p-values at the six different time points comparing the** ***ex vivo*** **and** ***in vitro*** **experiments.** The boxes are higher in the middle, reflecting the larger p-values and the fact that the gene expression patterns are more similar for time points three and four.

Taking all TPs into consideration using an alternative method that assesses calculated pairwise differences, 650 genes were found to be DE (FDR adjusted p-value < 0.01) between the *ex vivo* and *in vitro* samples. The majority of these genes (403; 62%) were annotated in the *P. knowlesi* genome database (Pk4 assembly) as hypothetical genes [1,35]. Of note, sequences representing 32 *SICAvar* gene IDs were shown to be differentially up-regulated at the ring stage, which corresponds to 38% of the 101 *SICAvar* gene oligonucleotides present on the array (Figure 6). These 101 oligonucleotides represent 99 unique *SICAvar* gene IDs plus two contigs with *SICAvar* gene sequences with most annotated at the time of this study as gene fragments [35]. Some have since been confirmed as contiguous sequences that are part of the same gene [30]; others occur next to gaps in the assembly [35]. Thus, the *SICAvar* oligonucleotides on the microarray do not represent the entire reported *SICAvar* gene family, which had been estimated at 107 or 108 members [29,35]. An updated *P. knowlesi* genome is forthcoming, which resolves many of these gaps (Chappell *et al.*, personal communication).

In contrast, only three out of 53 (5.6%) of *kir* gene family members represented on the array were identified as DE over all TPs. Unlike the *SICAvar* genes, the *kir* genes were not up-regulated preferentially in the ring stages. Two *kir* genes were identified as being DE at TP1 (rings), four at TP2 (early trophozoites), and two at TP6 (schizonts). The remaining DE genes include 31 ribosomal genes (4.5%) and various genes involved in either protein synthesis or processing.

### Genes differentially up-regulated *ex vivo*

A number of other genes were found to be specifically up-regulated in the *ex vivo* cultured parasites (Additional file 6, Panels 1–6). The majority of these were more highly expressed in the earliest and latest stages of development (Figure 6). This subset includes genes involved in proteolysis (PKH_011450, *proteasome subunit beta type* gene) and radical-based catalysis (PKH_144460, *radical S-adenosyl methyltransferase protein* gene), relevant for diverse metabolic reactions including biodegradation pathways. Interestingly, PKH_143910, the putative ApiAP2 transcription factor exhibited similar dynamics *ex vivo* and *in vitro*, but at a much higher level *ex vivo*, especially at earlier stages. Differential expression of genes involved in transcription in early stages has been documented before in *P. falciparum* [2,4]. Some unexpected transcripts were also detected such as the *P. knowlesi* homolog of *pfg377* (PKH_146780), which encodes a protein that is specifically found on female gametocytes, and the PSOP12 homolog (PKH_101880) that encodes a secreted protein at the ookinete stage. Gametocytes are often produced when the parasitaemia reaches high levels in rhesus macaques, and this could explain the detection of the *pfg377* homolog transcript. The ookinete protein could either have a separate function in the blood-stage parasites, or it is possible that the transcript is produced and maintained early, prior to its ultimate translation in the insect stage.

### Genes differentially up-regulated *in vitro*

Only a few genes were found to be up-regulated in the *in vitro* compared to *ex vivo*-cultured parasites (Additional file 6, Panels 6–8). Most of these genes were expressed at higher levels in the later stages of the IDC. Several genes involved in metabolism and cell growth were significantly up-regulated in the *in vitro* cultures: e.g., *porphobilinogen deaminase* (PBGD) (PKH_130850) and *protein tyrosine phosphatase* (PKH_122120). The former is involved in the haem biosynthesis pathway, catalyzing porphobilinogen to uroporphyrinogen III (UROGEN III) (EC 2.5.1.61) [1,46]. The host PBGD, also called hydroxymethylbilane synthase, is most highly expressed in the spleen and bone marrow in humans based on tissue-specific ESTs (Additional file 7) [47]. *Plasmodium* parasites utilize host haem synthesis proteins in their erythrocytic life cycle in addition to their own *de novo* pathway [48], however the host PBGD is much more efficient [46]. It could be expected, then, to see parasite PBGD up-regulated when the host enzyme is dysregulated, as in a splenectomized host, or in *in vitro* culture. Conversely, if erythropoiesis is stimulated in the bone marrow during malaria infection, host PBGD may become up-regulated, decreasing the requirement of parasite *de novo* synthesis. Finally, one *SICAvar* gene (PKH_020570) was highly up-regulated *in vitro* in the schizonts (TP6), and less so in the ring-stage (TP1 and TP2) samples. While this could mean that this *SICAvar* gene or encoded protein has a unique role for the parasite’s biology, the protein encoded by PKH_020570 was not identified in any blood-stage samples so far analysed by LC-MS/MS. It is also intriguing that this gene was also found to be up-regulated in *P. knowlesi* infected RBCs from a splenectomized animal (manuscript in preparation).

### Invasion and basic metabolic functions

The basic invasion, growth and multiplication mechanisms of *P. knowlesi* parasites *in vivo*, *ex-vivo* and *in vitro* are similarly efficient. In each case, within a similar time frame (25 to 33 hours), the merozoites effectively enter RBCs and transform through the trophozoite and schizont stages of development to produce progeny merozoites. As anticipated, transcripts representing genes encoding proteins known to be involved in the invasion of RBCs were similarly detected, reflecting common IDCs for both the *ex vivo* and *in vitro* parasite samples; e.g. *apical membrane antigen 1* [49], *merozoite surface protein 3* [50,51], *rhoptry associated proteins* [52], and *reticulocyte binding protein* homologs [53,54] (see Additional file 6 Panels 14–22). Merozoite surface protein 1 (PKH_072850) did exhibit an oscillating pattern over time in one of the *in vitro* replicate experiments. All in all, while similarities were noted in many such comparisons, it remains possible that subtle differences and unnoticed temporal factors may make a big difference in the expression of various genes and the timely function of the encoded proteins. Small or subtle differences in transcript patterns detected may have an impact on protein expression and function, but overall the data are consistent with the fact that the *ex vivo* and *in vitro*-derived parasites efficiently invade and grow in rhesus RBCs. The differences in the timing of the blood-stage cycles, likely simply reflect the adjustment of the parasites from the synchronization procedures. It is also worth noting that similar transcript levels were found for genes with various basic metabolic functions. For example, *14-3-3* and *actin* transcripts were detected at similar levels (Additional file 6, Panels 25–26). Genes annotated as encoding blood-stage membrane proteins and integral membrane proteins were also examined, and with the exception of the *SICAvar* gene family (discussed below), found no significant differences (Additional file 6, Panels 23–24).

### Epigenetic regulation

It is important to know whether *Plasmodium* genes that encode proteins involved in epigenetic regulation are differentially expressed *in vivo* compared to *in vitro* culture, especially given increasing knowledge of the importance of epigenetic regulation in *Plasmodium* [55-59], including the specific role of epigenetic regulation originally attributed to *var* gene expression in *P. falciparum* [60,61]. Overall, genes encoding histone and histone-modifying proteins exhibited similar levels of expression throughout the IDCs in the parasites grown *ex vivo* and *in vitro*. Interestingly, however, *sir2* transcripts (PKH_122390) were more abundant in the late stages of *ex vivo* parasites compared to *in vitro* (Additional file 6, Panel 13). Further analysis is required to determine if this difference in expression is critical for the *in vivo* expression of SIR2 in relation to the specific regulation of the *SICAvar* gene family and the up-regulated expression of many *SICAvar* transcripts in the ring-stage parasites *ex vivo*.

### *SICAvar* gene expression is dramatically down-regulated *in vitro*

**Figure 7:**
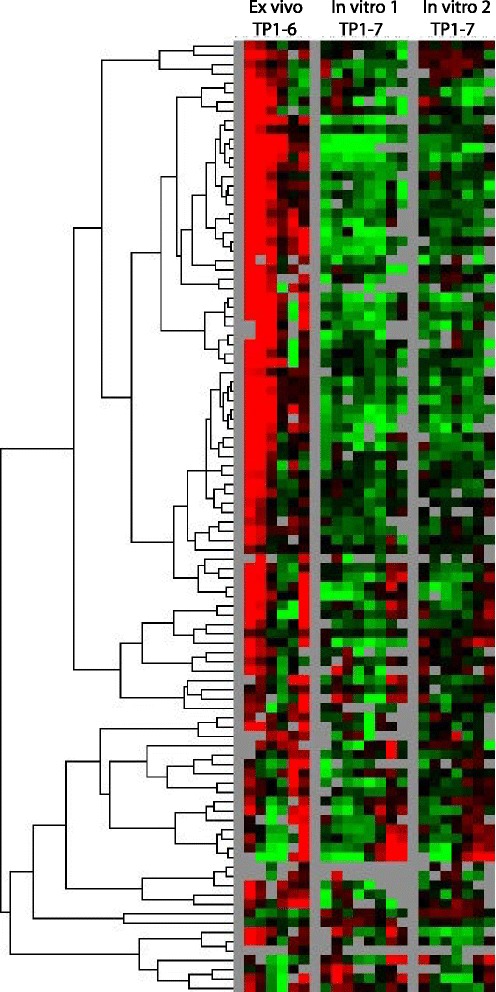
**Heat map of all** ***SICAvar*** **genes in the** ***ex vivo*** **and two** ***in vitro*** **experiments, based on normalized intensity values.**

The normalized intensity values of *SICAvar* genes were inputted into the Cluster program and visualized with TreeView (Figure 7). These data clearly show that the *SICAvar* gene family as a whole is down-regulated when the iRBCs are grown *in vitro.* In the *ex vivo* culture, the *SICAvar* gene IDs with the highest level of transcript expression demonstrated by the microarrays are PKH_050020 and PKH_072700/PKH_072710 (the latter two gene IDs represent a single *SICAvar* gene). These gene IDs were identified by LC-MS/MS for the *P. knowlesi* clone known as Pk1(A+), from which the *P. knowlesi* microarray oligonucleotides were based, but not for the Pk1(B+)1+ clone [30]. Also detected at a high level was PKH_113950, which is highly expressed in the Pk1(B+)1+ clone [30]. However, a recent BLAST search revealed that the PKH_113950 oligonucleotide probe hits another *SICAvar* gene, PKH_020270, with 100% identity.

It was also determined if SICA proteins could be detected in the *in vitro* cultured parasites using LC-MS/MS and found only one unique peptide corresponding to one SICA protein (PKH_030540); this contrasts to a high level of expression of multiple SICA proteins in established clones from rhesus macaque infections [30]. These findings are comparable to what is observed when *P. knowlesi* SICA[−] blood-stage parasites are grown in a splenectomized rhesus monkey, in which case neither functional full-length *SICAvar* transcripts nor SICA proteins are observed [30]. It has been known that the presence of the spleen is necessary for the expression and switching of SICA phenotypes *in vivo* [34], and that the spleen also has an apparent role in the expression of PfEMP1 encoded by the *var* genes and cytoadherence [62-64]. Whether only host splenic factors are critical for the expression of the *SICAvar* gene family or also other host influences is unknown. Similar questions remain valid with regards to the expression of the *P. falciparum var* genes *in vivo*. The stage-specific *ex vivo* microarray data are consistent with published expression library screening and standard RT-PCR experiments showing that many *SICAvar* genes are expressed as transcripts in the ring stage of development, without any evidence for the predominant transcription of any particular *SICAvar* transcript(s) [28-30,65].

One possible interpretation of these microarray data with regard to antigenic variation is that the default state for *SICAvar* genes in *P. knowlesi* is ‘off’ , and that there are host cues that turn them ‘on’. The spleen is one predicted source of these cues, since it has been shown that SICA protein expression at the surface of iRBCs is lost after passage in splenectomized hosts [34], and it has been shown quantitatively that the level of *SICAvar* transcripts detectable in splenectomized hosts is minimal in comparison with intact hosts. Moreover, full-length *SICAvar* transcripts are not detected in RNA extracted from SICA[−] parasites [30].

## Conclusions

This stage-specific microarray analysis shows a marked difference in gene expression between *P. knowlesi* blood-stage parasites derived from a rhesus monkey infection and *P. knowlesi* parasites adapted for *in vitro* culture. Most strikingly, expression of the *SICAvar* variant antigen genes, which are related to the *P. falciparum var* genes and associated with increased virulence *in vivo* [28,31,34] is up-regulated in the *ex vivo* cultures. Thus, *in vitro* cultures can provide a negative phenotype (in the “off state”) for *SICAvar* gene expression in comparison with *ex vivo* samples (in the “on state”), similar to what has been demonstrated in splenectomized hosts [30,34,63,66]. *Ex vivo* cultures would therefore be important for unraveling the molecular mechanisms that control *SICAvar* gene expression and switching. Further investigations are required to evaluate what other genes may be implicated in the regulation of the *SICAvar* and potentially *var* gene family in the context of the *in vivo* host environment. Subsequent studies (Lapp, Chappel *et al.*, personal communication) using RNA-Seq technologies will provide useful comparisons to expand upon the findings reported here.

Other clear examples of up-regulated and down-regulated genes were also detected between the *ex vivo* and *in vitro P. knowlesi* samples, which should be taken into consideration when aiming to understand the parasite’s natural programmed growth and development with the inherent influence of host factors *in vivo*. Critically, the data reported here bring emphasis to the fact that host-derived biological cues are important for *Plasmodium* gene expression.

## Additional files

Additional file 1:
**MA oligos.** List of gene IDs and oligo sequences used in the analysis. Additional file 2:
**Workflow.** Data analysis workflow. Additional file 3:
**PK4165genes_vivo_vitro.** Data table of raw and centered intensities of the genes used for analysis. Additional file 4:
**DE by stage.** Differential expression of genes in *ex vivo* and *in vitro* by time point. Additional file 5:
**Phase.** Phase correlation between *ex vivo* and the two *in vitro* time courses. Additional file 6:
**Panels showing comparative gene expression dynamics between** ***ex vivo*** **and** ***in vitro*** **time courses.**
Additional file 7:
**ESTs of HMBS.** Tissue-specific expression of HMBS in human.
