# Supplementary figures and images for: Plasmodium knowlesi gene expression differs in ex vivo compared to in vitro blood-stage cultures
Source: Malar J. 2015 Mar 13;14:110. doi: 10.1186/s12936-015-0612-8 (PMC4369371; doi:10.1186/s12936-015-0612-8)

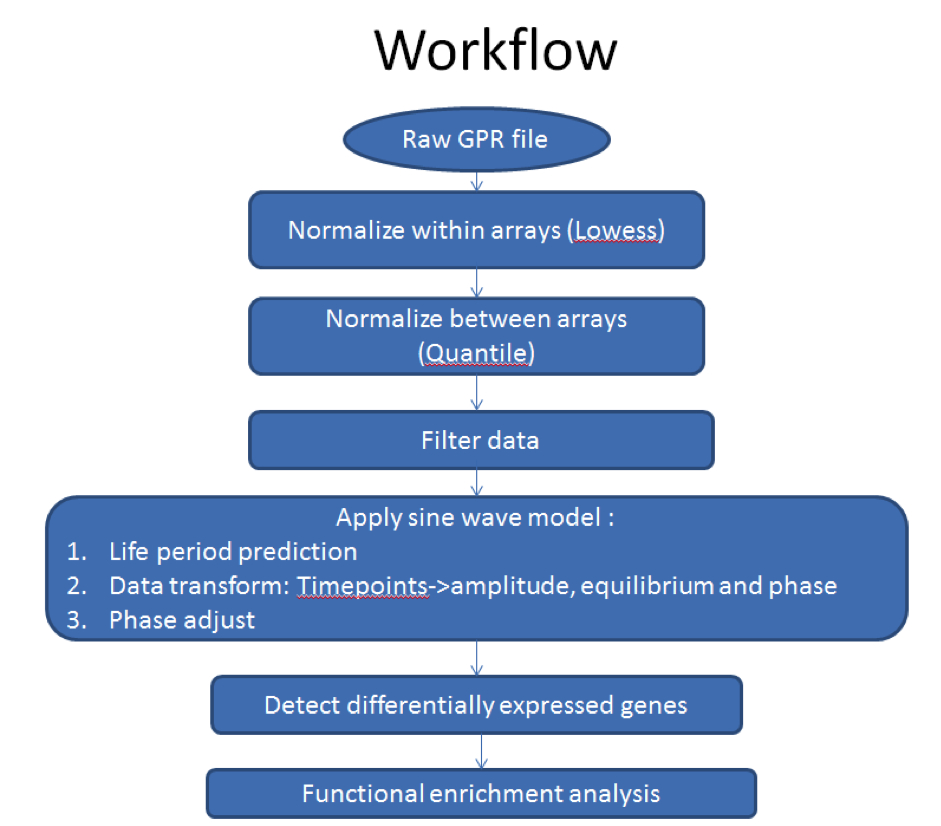

Supplement: Additional file 2: — Workflow. Data analysis workflow. [file 12936_2015_612_MOESM2_ESM.png]

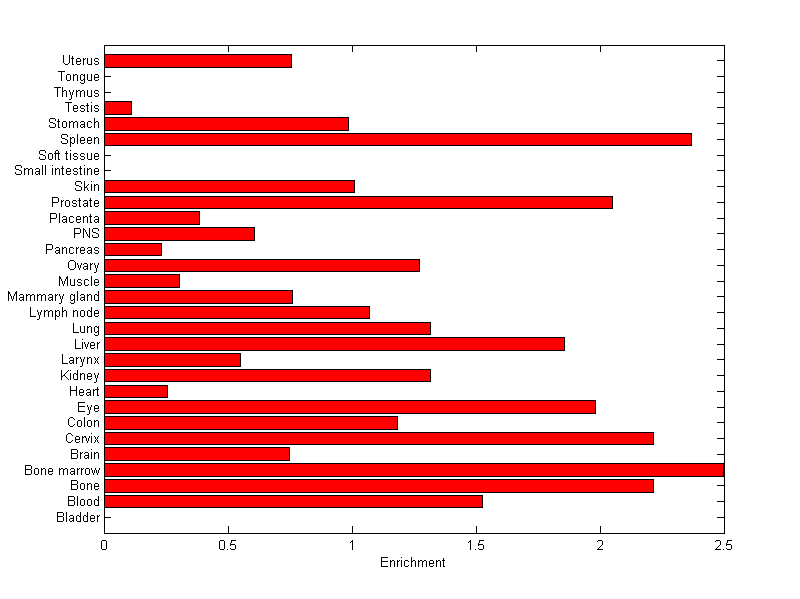

Supplement: Additional file 7: — ESTs of HMBS. Tissue-specific expression of HMBS in human. [file 12936_2015_612_MOESM7_ESM.tiff]
